# Optimizing the Rheological and Thermal Behavior of Polypropylene-Based Composites for Material Extrusion Additive Manufacturing Processes

**DOI:** 10.3390/polym15102263

**Published:** 2023-05-11

**Authors:** Giulia Bernagozzi, Daniele Battegazzore, Rossella Arrigo, Alberto Frache

**Affiliations:** 1 Department of Applied Science and Technology, Politecnico di Torino, Viale Teresa Michel 5, 15121 Alessandria, Italy; giulia.bernagozzi@polito.it (G.B.); daniele.battegazzore@polito.it (D.B.); rossella.arrigo@polito.it (R.A.); 2 Local INSTM Unit, 15121 Alessandria, Italy

**Keywords:** polypropylene, 3D printing, material extrusion additive manufacturing, rheology, mechanical properties

## Abstract

In this study, composites based on a heterophasic polypropylene (PP) copolymer containing different loadings of micro-sized (i.e., talc, calcium carbonate, and silica) and nano-sized (i.e., a nanoclay) fillers were formulated via melt compounding to obtain PP-based materials suitable for Material Extrusion (MEX) additive manufacturing processing. The assessment of the thermal properties and the rheological behavior of the produced materials allowed us to disclose the relationships between the influence of the embedded fillers and the fundamental characteristics of the materials affecting their MEX processability. In particular, composites containing 30 wt% of talc or calcium carbonate and 3 wt% of nanoclay showed the best combination of thermal and rheological properties and were selected for 3D printing processing. The evaluation of the morphology of the filaments and the 3D-printed samples demonstrated that the introduction of different fillers affects their surface quality as well as the adhesion between subsequently deposited layers. Finally, the tensile properties of 3D-printed specimens were assessed; the obtained results showed that modulable mechanical properties can be achieved depending on the type of the embedded filler, opening new perspectives towards the full exploitation of MEX processing in the production of printed parts endowed with desirable characteristics and functionalities.

## 1. Introduction

Fused filament fabrication (FFF), also known as fused deposition modelling (which is the original trademark by Stratasys), is one of the most used 3D printing techniques for thermoplastic polymers [1]. FFF belongs to the material extrusion (MEX) additive manufacturing processes because this method involves the construction of a desired object through the layer-by-layer deposition of a molten thermoplastic filament extruded through a nozzle [2,3,4]. In recent years, FFF has been increasingly used more frequently compared to other 3D printing technologies for polymers as it is quite a simple and inexpensive process that allows the obtainment of complex geometries with a very high degree of customization [5,6]. Due to these advantages, FFF is steadily shifting from a technique mainly used for rapid prototyping towards an effective production method for the formulation of functional and high-performance parts to being potentially suitable for several applications in advanced sectors, such as medicine or the automotive field [7]. Nevertheless, FFF presents the typical limitations of additive manufacturing processes, mainly related to the difficult achievement of economies of scale due to the higher cycle time with respect to conventional processing technologies such as injection molding and the need for post-processing operations to remove the support structures (which are imperative for the achievement of complex design) [4]. These things considered, parts obtained through FFF are usually characterized by inferior mechanical properties due to the weak interlayer adhesion, which is generally achieved through the layer-by-layer construction and inherent porosity [8,9]. Additionally, a further weakness of FFF relies on the lack of an extended portfolio of suitable materials [10,11]. Commonly, amorphous or low-crystalline polymers (such as poly(lactic acid) and acrylonitrile-butadiene-styrene) are used as feedstocks for the formulation of filaments for FFF processes, mainly due to the low degree of shrinkage exhibited by these polymers during solidification, leading to the obtainment of objects characterized by a high degree of accuracy [12,13]. Conversely, the crystallization undergone by semi-crystalline thermoplastics causes a large degree of volumetric contraction, resulting in the warpage and shrinkage of printed parts [14,15]. This last issue is even more exacerbated for polymers, such as polypropylene (PP), showing rapid crystallization, in which the crystallites formed during the solidification step hinder the chain diffusion across the deposited layers, further reducing the interlayer adhesion, hence negatively affecting the final mechanical properties of the obtained object [16].

It should be taken into consideration that the successful realization of FFF processing is strongly influenced by the rheological behavior of the thermoplastic filament [17,18]. In fact, as widely documented in the literature, a material processable through FFF should be able to be extruded through the printing nozzle, then the extruded material should retain its shape in the standoff region (between the nozzle and the printing bed) and, finally, the deposited molten filament must remain geometrically stable during and after the deposition step [19,20,21]. All these requirements are satisfied if the material presents a non-Newtonian rheological behavior involving low viscosity values at a high shear rate (in order to ensure its flowability during the extrusion through the nozzle) and a rapid increase in the viscosity at quasi-zero shear conditions (guaranteeing shape-stability of the extruded filament during and after the deposition, while avoiding oozing phenomena during the printing stage) [22].

In a previous work [23], we successfully developed a PP-based material suitable for FFF processing, demonstrating that the introduction of 20 wt% of talc in a heterophasic PP copolymer is beneficial for minimizing the melting enthalpy of the polymer matrix, allowing one to overcome the issues related to the typical high volumetric shrinkage of PP. On the other hand, the concurrent effect of the embedded fillers and the matrix copolymerization induced a significant modification of the PP rheological properties, leading to the obtainment of a well-developed non-Newtonian behavior that, as stated before, is the strictest criterion for classifying a thermoplastic as FFF-printable.

Quite recently, some authors focused on the modification of PP through the introduction of different fillers, such as glass fibers [24,25,26], glass spheres [26,27,28], expanded spherical perlite [29], clay [30,31,32], alumina [33], and talc [26], in order to obtain FFF processable materials. In this context, Winter et al. [26] formulated PP-based composites containing glass fibers, glass spheres, and talc suitable for FFF, showing that the introduction of increasing contents of the fillers favorably modified the thermal properties of the polymeric matrix, reducing the final warpage of the printed parts. Among the different exploited fillers, glass fibers resulted in the obtainment of reduced final warpage compared to glass spheres and talc due to the preferential orientation of the anisotropic fillers achieved during the printing process.

Despite the development of FFF printable PP-based materials attracting a steadily increasing interest in recent years, most of the research works reported in the literature mainly evaluate the effect of the presence of different fillers and/or of processing parameters on the final properties of the printed parts, without a systematic assessment of the influence of different fillers on the modification of the polymer properties for properly designing a FFF printable PP-based material.

In this study, composites based on a heterophasic PP copolymer containing different contents of micro-sized (namely, calcium carbonate, talc and silica) and nano-sized (i.e., an organo-modified nanoclay) fillers characterized by different shapes, average dimensions, and aspect ratios were obtained through melt compounding. The thermal properties and rheological behavior of the resulting materials were evaluated, with the aim of optimizing the material formulation for FFF processing. The composites showing the best performance in terms of FFF processability were then scaled-up in a twin-screw extruder and further processed (after a close optimization of the process parameters) to obtain printable filaments and 3D-printed parts. The assessment of the morphology of the filaments and of the FFF printed samples allowed us to correlate the processability of the selected PP-based composites with the influence of the different fillers on the matrix’s rheological and thermal behavior. Finally, the assessment of the mechanical properties of the printed materials demonstrated the possibility of achieving modulable mechanical characteristics depending on the type of embedded fillers.

## 2. Materials and Methods

Firstly, a preliminary study on different PP-based composites (produced by means of a mini-extruder with a recirculation channel with processing parameters specified in Section 2.2.1) was carried out. The prepared materials were characterized from a rheological and thermal point of view in order to evaluate the most suitable composites for 3D printing.

Some of the composites studied in the preliminary phase were then selected to scale-up the process in a laboratory twin-screw extruder (with process parameters specified in Section 2.3). Finally, the filaments fabricated with extruded materials were used to 3D print specimens for tensile characterization.

### 2.1. Materials

In this study, ISPLEN^®^ PB 170 G2M (supplied by Repsol–Chemicals, Madrid, Spain) was used. It is a PP heterophasic copolymer with density of 905 kg/m^3^ and MFR of 12 g/10 min.

Micro-fillers added to PP were talc, calcium carbonate, and silica; a lamellar phyllosilicate was used as nano-filler. Talc HTP1 grade (mean diameter: 1.9 µm) was supplied by IMI Fabi Spa (Valmalenco, Italy). Calcium carbonate OMYACARB^®^ 1T-AV grade (mean diameter: 2 µm) was supplied by OMYA (Oftringen, Switzerland). Silica particles (diameter in the range 10–20 µm to 0.5–2 µm) were obtained from the rice husk supplied by S.P. Spa through a process of calcination of the husk itself. The lamellar phyllosilicate Cloisite^®^ 20 was a bentonite modified with bis(hydrogenated tallow alkyl)dimethyl salt and supplied by BYK (Wesel, Deutschland). To enhance the dispersion of the nano-filler within the matrix, it was added to a compatibilizer, i.e., PP grafted with maleic anhydride (0.6 wt%), supplied by Sigma-Aldrich (Darmstadt, Deutschland).

### 2.2. Processing

#### 2.2.1. Preliminary Study

PP-based composites were processed in a twin-screw mini-extruder Xplore MC 15 (DSM, Sittard, The Netherlands), which is a 15 mL compounder with a recirculation channel allowing the material to be kept inside the chamber throughout the process. PP and fillers were manually fed all together into the mini-extruder. The heating temperature was set at 200 °C. The screw speed was set at 50 rpm for the feeding time and increased up to 100 rpm for the residence one, fixed at 5 min. Samples for the rheological tests (having diameter = 25 mm and thickness = 1 mm) were obtained through a compression molding step using a hot plate press Collin P 200T (Maitenbeth, Germany) operating at 200 °C under a pressure of 100 bar for 2 min. The thermal analyses were carried out directly on the extrudates.

### 2.3. 3D Printing Process

3D printing-suitable PP-based composites were processed inside an extruder Process 11 (Thermo Fisher Scientific, Waltham, MA, USA). The extruder had two co-rotating screws (11 mm diameter) placed inside a cylinder characterized by 7 heated zones. The screw speed was set at 400 rpm and the heating temperature profile was set at 190 °C for all the zones. Two volumetric feeders (MT and RT model from Brabender, Duisburg, Deutschland) were used: the first, for the polymer, at the beginning of the extruder; the second supplied filler powder and was placed about halfway through the barrel. At the exit of the extruder, there was a tank containing water for cooling the extruded, which was then pelletized with a rotating cutter (Varicut pelletizer 11 mm).

Next, a 1.0 Advanced filament making machine by 3Devo (Utrecht, The Netherlands) was used to produce a filament with a nominal diameter of 1.75 mm. The process used was the same as described in a previous article [23]. The parameters for filament fabrication were optimized and will be discussed in Section 3.2.1.

Roboze One 3D printer (Bari, Italy) equipped with a 0.4 mm nozzle was used to prepare tensile test specimens (ISO 527 standard type 5A) with the help of Simplify 3D software to set the parameters. As PP shows serious issues when adhering to any surface, the 3D printer bed was fully covered with a PP surface. The specimen was placed for each material in the XY plane. The raft was used because it improves the adhesion of the part to the printing plate and prevents defects in the sample removing. 3D printing parameters were also optimized. Some settings were kept unchanged for all of the specimen preparation: infill percentage of 100%, deposition pattern ±45°, extrusion width of 0.4 mm, layer thickness of 0.2 mm, extrusion temperature of 260 °C, and extrusion speed at 30 mm/s.

### 2.4. Characterization Techniques

The thermal properties of the formulated materials were evaluated by Differential Scanning Calorimetry (DSC) using a DSC Q20 supplied by TA Instruments (New Castle, DE, USA). Each sample was put into a controlled chamber with nitrogen and heated from −50 °C to 220 °C at a heating rate of 10 °C/min. The information achieved by this analysis was that melting enthalpy (ΔHm) could be evaluated as the area under the exothermal peak of the heat flow. ΔHm values, in fact, can be indicators of the degree of crystallinity developed in the printed part and reflect the volumetric shrinkage of the material [34,35].

The rheological behavior of unfilled PP and all formulated composites was evaluated using an ARES (TA Instrument, USA) strain-controlled rheometer in parallel plate geometry (plate diameter = 25 mm). Preliminary strain sweep tests were carried out at 200 °C and ω = 10 rad/s. The complex viscosity and storage and loss moduli were measured by performing frequency scans at 260 °C from 10^2^ to 10^−1^ rad/s. The strain amplitude was selected for each sample in order to be within the linear viscoelastic region.

The surface morphology and section of filaments were investigated using a EVO 15 Scanning Electron Microscope (SEM) from Zeiss (beam voltage: 20 kV working distance: 8.5 mm, Oberkochen, Germany). The sections were investigated on small pieces of filaments by fracturing them into liquid nitrogen and then covering them with a sputtered gold layer. Quantitative compositional information of sections of filaments was investigated by SEM, which was made possible through Energy dispersive X-ray Spectroscopy (EDS), which provides a spectrum with peaks related to the elements in the sample with peak amplitude proportional to the amount.

Tensile tests were performed at room temperature using an Instron^®^ 5966 (Norwood, MA, USA) equipped with 2 kN pneumatic grips and a gauge length of 50 mm. The crosshead speed was set, for all the specimens, at 1 mm/min in the first part of the test to accurately calculate the elastic modulus, which was equal to 10 mm/min once a deformation of 0.25% was exceeded, in order to bring the specimen to break faster. Five specimens were used for each formulation, and the average values and corresponding standard deviations of the tensile modulus (E), elongation at break (ε), and maximum tensile strength (σ) were calculated and reported.

## 3. Results

### 3.1. Preliminary Study

The preliminary study consisted of determining the most suitable PP-based formulations for the FFF process. All the composites with different types and contents of fillers were processed in the mini-extruder. Then, each composite was characterized through thermal and rheological analysis in order to find the best 3D printable formulations.

The fillers added to PP had different aspect ratios: talc and silica are lamellar fillers, while calcium carbonate has a lower aspect ratio and a more circular shape. With this in mind, a nano-filler with a lamellar shape was added to PP. Micro-composite materials were fabricated at three different filler concentrations, i.e., 10 wt%, 20 wt%, 30 wt%, whereas nano-composites were fabricated at 1 wt% and 3 wt%. The codes and formulations of extruded composites are displayed in Table 1.

**Figure 1:**
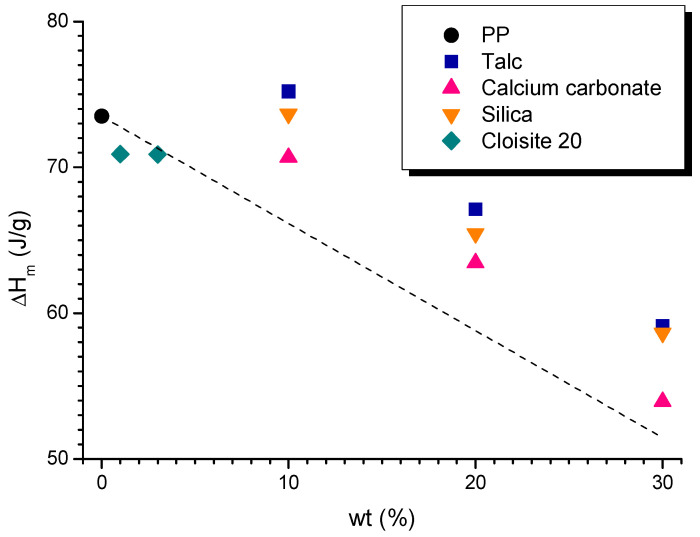
Melting enthalpy values (ΔHm) as a function of the content of filler for PP and PP-based formulations.

The first requirement that a material must fulfil to be 3D printable is to have a low content of crystallinity, to avoid phenomena related to volumetric shrinkage during the material solidification, inducing the detachment of the part from the printing bed during the process [24]. However, in order to find the most suitable materials for the 3D printing process, the trend of the melting enthalpy (ΔHm) as a function of the loading of filler was evaluated (Figure 1). ΔHm should decrease to facilitate a decrease in the fraction of the crystalline phase, consequently leading to lower shrinkage [35]. Compared to neat PP, the addition of 10 wt% of silica does not cause a remarkable decrease in the ΔHm value; otherwise, the introduction of 10 wt% of talc promotes an incremental increase this value. On the other hand, considering 10CC, the ΔHm value decreases (−4%). With a further increase in the loading of the filler to 20 and 30 wt%, ΔHm values decrease for all composites. More specifically, 30CC showed the highest decrease in ΔHm value compared to the unfilled PP (−27%), whereas for 30T and 30S, ΔHm decreased by 20%. Similar results implying a decrease in melting enthalpy values when increasing the amount of filler have been observed by other authors [26,36,37,38].

Compared to the dotted line in Figure 1, which represents the theoretical trend of ΔHm of PP considering the amount of matrix within each composite, all formulations with micro-fillers show a higher value of ΔHm. This indicates that the fillers act as nucleating agents. The higher values of melting enthalpy of the composites with talc, that is, a lamellar filler with a greater contact area, therefore the crystallinity is more induced. Regarding the nano-filler, both 1Cl and 3Cl have a similar ΔHm value, which decreased by about 4% compared to neat PP, as did 10CC. Other authors found that, when adding graphene nanoplatelet to PP, the ΔHm value does not vary according to the amount added inside the nano-composite [39]. Additionally, the same result was obtained by Fuad et al. in PP/calcium carbonate nanocomposites [40].

**Figure 2:**
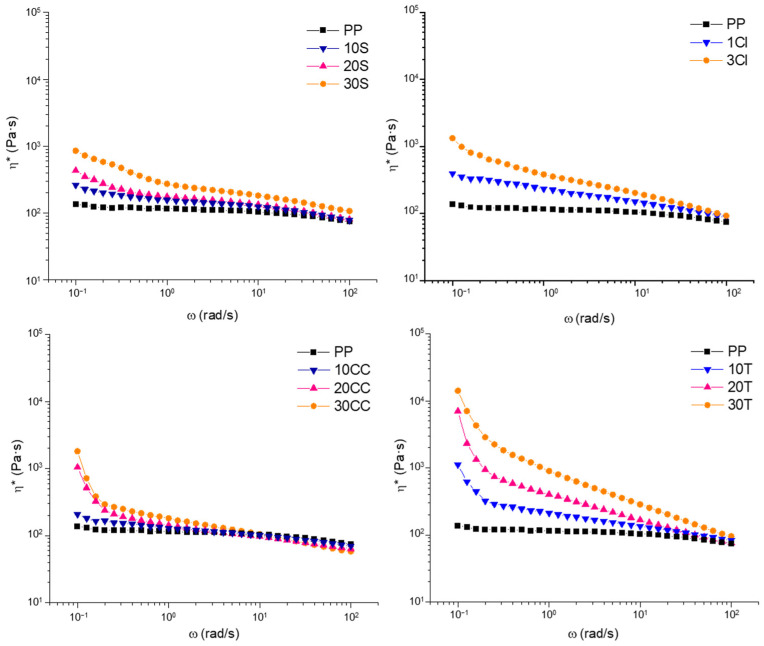
Complex viscosity curves collected at 260 °C for unfilled PP and all formulated composites.

The second design requirement that makes a material suitable for the 3D printing process is dictated by the rheological behavior. In fact, it is necessary that the material presents remarkable non-Newtonian features, including a pronounced yield stress together with a strong shear thinning behavior [23,41]. The latter is required to ensure the low viscosity of the material during the flow through the nozzle at high shear rates. Otherwise, yield stress is preferred when zero-shear conditions occur, i.e., when the materials exit the nozzle, because a rapid increase in the viscosity is needed to avoid dripping phenomena and ensure shape stability [22]. Figure 2 reports the trends of the complex viscosity (η*) as a function of the frequency (ω) for all investigated materials. PP shows a Newtonian behavior at low and intermediate frequencies, followed by a mild shear thinning at high frequencies. Upon adding fillers, all materials exhibit non-Newtonian characteristics at low frequencies, with an increase in complex viscosity when the frequency decreases and a more pronounced shear thinning compared to the neat PP. This behavior becomes progressively more pronounced with increasing filler loading. The observed disappearance of the Newtonian plateau is attributable to the presence of the embedded solid fillers, which, by interacting with the polymer network composed of entangled macromolecules, hinder the complete relaxation of the polymer chains. To ensure the extrudability of the materials, shear thinning behavior is required; therefore, among the characterized materials, the most suitable for the 3D printing process are those with the highest percentage (by weight) of filler. As already stated, another requirement is the presence of yield stress behavior. From a general point of view, it can be observed that, by increasing the amount of fillers, the yield stress increases; however, significant differences emerge when considering the effect of the aspect ratio and shape of fillers on the matrix’s rheological behavior. More specifically, the yield stress behavior is more pronounced for the composites containing talc, and in this case, the introduction of 10 wt% of fillers is enough to induce significant variations in the matrix’s behavior. A similar trend is shown by the composite containing calcium carbonate, notwithstanding the higher loadings of fillers required. Differently, the introduction of silica particles does not remarkably affect the low-frequency viscosity of the polymer matrix, as testified by the absence of a well-developed yield stress behavior. Concerning the nanocomposite, it is interesting to observe that the addition of only 3 wt% of cloisite leads to yield stress behavior and viscosity values at low frequencies comparable to those of 30CC, which has ten times the amount of filler.

The differences between the rheological behavior of the composites can be attributed to the different aspect ratios of the fillers, affecting the specific physical filler–filler and filler–polymer interactions established in the composite systems. In particular, calcium carbonate and silica, characterized by a low aspect ratio, are not able to significantly alter the dynamics of the polymer chains when embedded within the matrix; therefore, high loadings of these fillers are required to observe significant variations in the rheological behavior of the matrix [22]. Additionally, it should be considered that the used silica (obtained from rice husk) is characterized by a wide particle distribution [42] and has a porous structure; both of these features could further worsen the establishment of effective polymer–filler interactions. Specifically, fillers with a high aspect ratio, such as talc, and to a greater extent, the nanofillers, are more likely to create a network spanning the whole composite, significantly affecting the relaxation dynamics of polymer chains and thus the low-frequency rheological behavior. In particular, in the case of the nanocomposites, the appearance of a more pronounced shear thinning and yield stress in 3Cl instead may reflect a good dispersion and the exfoliation of the nano-filler inside the matrix, as other authors have already observed [32,43].

As a consequence of the previous considerations about the thermal and rheological characterizations, it was decided to proceed with the filament fabrication and subsequent 3D printing of three of the analyzed composites: 30T, selected for the 3D-printing-suitable rheological behavior characterized by both shear thinning and yield stress; 30CC for thermal properties because of the lower ΔHm value compared to other composites, which should lead to less shrinkage; and 3Cl to evaluate the 3D printability of a PP-based nanocomposite due to a lack of results in the literature [30,31,32], especially in comparison with other PP-based composites.

### 3.2. 3D Printing Process

After the preliminary study, the selected composites were melt compounded in a co-rotating twin-screw extruder Process 11. Then, the pellets of each composite were used to feed the Next 1.0 Advanced instrument in order to obtain a filament with the appropriate features needed to be 3D printable.

#### 3.2.1. Filament Fabrication

Filament fabrication is a fundamental step for the 3D printing process. It is a continuous process that cannot undergo interruptions due to, for example, irregularities in the filament. A filament suitable for FFF must have the following characteristics: constant diameter equal to 1.75 mm; round shape; and a smooth surface with low roughness. In order to optimize the processing conditions to achieve a regular filament, the filament fabrication went through a series of steps that involved changing the three main parameters of the filament extruder, namely temperature profile, screw speed, and fans speed percentage. The optimization of the parameters for the production of the filament started, for each material, from the selection of the temperature and then continued by modifying the remaining parameters to obtain the required characteristics.

For every composite, it was decided, first and foremost, to set the temperature of extrusion. However, in general, a temperature that is too low could lead to the excessively high viscosity of the material coming out of the nozzle, with the risk of this being that the filament cannot be expanded properly to reach the desired diameter. In these circumstances, the phenomenon of filament buckling may occur, during which the material is not stiff enough to withstand the applied pressure during the passage of the filament inside the nozzle [22,44]. Conversely, a temperature that is too high could result in an insufficient viscosity and uncontrolled flow of material coming out of the nozzle, which could undergo non-homogeneous solidification. On the other hand, the temperature profile is related to the screw speed—if the material coming out of the nozzle does not have the correct viscosity to be able to flow easily and reach the desired diameter, it may be necessary to increase the rpm of the screw. However, the screw speed was not immediately adjusted when changing its value, and for each filament, the screw speed was set to 2 rpm and gradually increased. In fact, it has been observed that a high speed causes an excessive amount of material to exit from the nozzle and does not allow any control of the filament diameter. Cooling fans can instead be set to a percentage value between 0 and 100%, and their greater influence on the filament implies a change in its shape. In fact, it is important to check the correct position of the fans to allow the filament to solidify at the top of the instrument. It has been observed that, if the fans flow air towards a direction lower than the ideal point, the filament obtained is characterized by a wavy shape. For each filament, a diffuser specially designed to allow for uniform cooling along the entire circumference of the filament and mitigate the fact that the filament has an oval shape was deployed, as already used and described by other authors [23,41].

The optimized process parameters that have led to filaments with the required characteristics are reported in Table 2.

**Figure 3:**
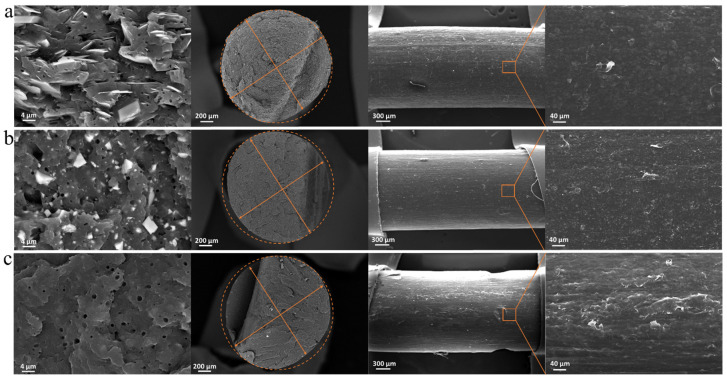
SEM images of section (HDBSD—back scattered) and surface (SE—secondary electron) of filaments of (**a**) 30T, (**b**) 30CC, and (**c**) 3Cl.3.3. FFF 3D Printing.

To evaluate filament diameter constancy, roughness, and circularity, SEM observations were performed, and some representative micrographs are shown in Figure 3.

The SEM micrographs of 30T’s filament section (Figure 3a) show an internal morphology characterized by good dispersion and good distribution of the filler lamellae in the polymer matrix. Furthermore, talc lamellae appear preferentially oriented in the flow direction. It is also possible to identify both voids and whiter parts with a circular shape of about 1 µm. The spherical inclusions can be related to the presence of polyethylene or ethylene propylene rubber particles, usually embedded in PP heterophasic copolymers. SEM images with a similar morphology have been reported in the study of Marcilla et al., where a PP copolymer from Repsol was used [45]. The morphological inspections carried out through SEM allowed the identification of a minimum and maximum diameter of 1.65 and 1.76 mm (highlighted by the arrows drawn in Figure 3), respectively, resulting in a not perfectly circular section. The SEM image of the 30T filament surface shows a diameter variability of 1% in the segment considered and a low roughness.

Considering the SEM image of the 30CC filament section (Figure 3b), as in the case of talc, a homogeneous distribution and dispersion of calcium carbonate within the polymer matrix was observed. Due to the nature of the copolymer used as the matrix, quasi-spherical particles and voids (attributable to the fingerprint of polyethylene or ethylene propylene rubber particles detached from the matrix during the brittle fracturing in liquid nitrogen performed for the observation of the samples through SEM) were again found. The filament section is not perfectly circular, as visible from the dashed circumference superimposed with a minimum and maximum diameter of 1.64 and 1.71 mm, respectively. With regard to the surface of the 30CC filament, a diameter variability of 2% and a low roughness comparable with 30T were found.

From the SEM image of the fracture surface of the 3Cl filament (Figure 3c) at the same magnification of the other two filaments, it is not possible to observe the presence of the embedded fillers. This may be an indicator that the nano-filler is well distributed and that no micrometric aggregates remained inside the material, owing also to the action of the added compatibilizer [46]. Therefore, the distribution of Closite20 is sub-micrometric. The presence of the nano-filler was confirmed by the Energy Dispersive X-ray Spectroscopy (EDS) via SEM. In Figure S1, Al and Si mapping of SEM images of 3Cl are reported, with a table reporting the weight fraction of all the elements detected. The Al and Si mapping suggests that Closite20 is well dispersed within the PP matrix. Once again, in Figure 3c it is worth noting that the section is not completely circular and a minimum and maximum diameter of 1.62 and 1.76 mm, respectively, were measured. The SEM image of the 3Cl filament surface shows a diameter variability of 2% and a more noticeable roughness than 30T and 30CC filaments.

In a similar way to the filament production, parameter optimization trials were also carried out for the 3D printing process. The parameters were chosen after observing the printed parts by evaluating compliance with geometric tolerances, the ability to reproduce details, and the presence of distortions and defects not foreseen in the CAD drawing. The main process parameters were discussed in Section 2.3. The only difference lies in the temperature set for the printing bed. For 30T and 30CC, the bed temperature was set at 50 °C. For 3Cl, the bed temperature was increased up to 70 °C as adhesion problems were encountered at 50 °C. In particular, at a lower temperature, in the first layers deposited of 3Cl, the two adjacent layers attached to each other, while at a higher temperature, this defect did not occur.

With regard to the 3Cl surface finish, the SEM images showed a rougher filament compared to 30T and 30CC, but upon observing the printed specimen, this aspect did not significantly influence the realization of the objects using FFF, and a satisfactory quality was obtained.

Each specimen was made with two external perimeters, which played significant roles in serving as boundaries for the filling. Commonly, the number of perimeters is set between 1 and 3. In terms of mechanical properties, the number of perimeters should help distribute the load more effectively across the perimeters as a result of a stronger interfacial bonds between deposited filaments and the denser structure [47].

**Figure 4:**
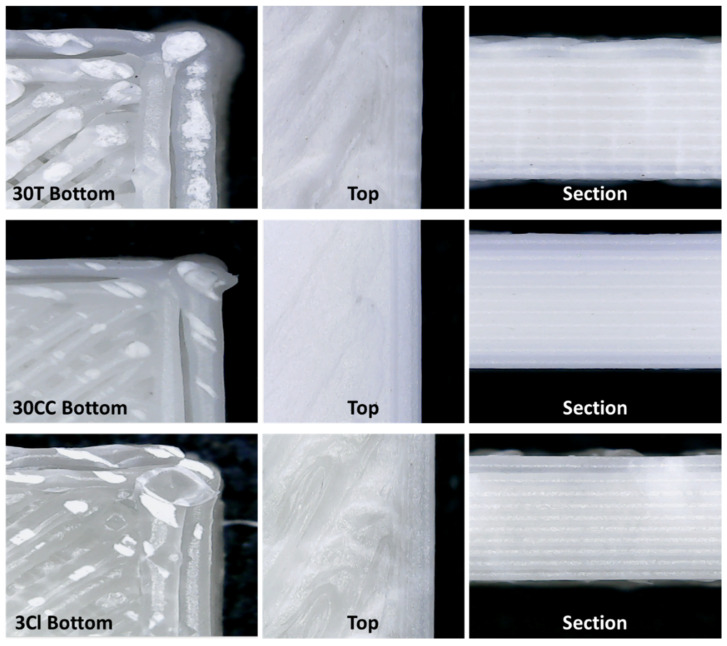
Optical micrographs of 3D-printed specimens for tensile tests.

Figure 4 shows the optical micrographs taken on the 3D-printed samples, which were obtained using the optimized process parameters listed above. The bottom of the part directly in contact with the raft is shown on the left. In the center, the top part of the specimen (i.e., the last layer) is shown, and the side section on the right is as well. It is evident that the upper and lower surfaces are different from each other. More specifically, in the lower part, the layers are clearly visible, probably because of the rapid solidification of the first layers deposited on the raft. On the other hand, the top layers are deposited on layers at a higher temperature; therefore, the spaces between the adjacent filaments are filled. In the thermal monitoring of the FFF process, Vaes D. et al. found that the temperature profile during deposition of the layers is influenced by both the conduction originating from the printing bed and the room air temperature [34]. The difference between the top and bottom of the specimen can probably be reduced by increasing the printing bed temperature. It is possible to notice the detachment of the outermost perimeter from the innermost one. This defect was resolved by proceeding from the bottom upwards until reaching the last layer, which is completely full.

**Figure 5:**
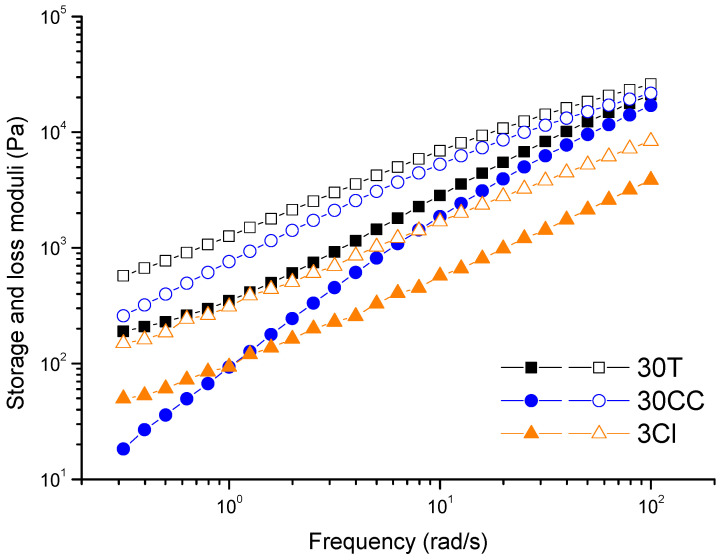
Dynamic moduli as a function of frequency for PP-based materials (G′ full symbols, G″ empty symbol).

The 10 deposited layers to produce the specimens for the subsequent tensile characterization are visible in the pictures of the side section. In the case of 30T and 3Cl samples, the upper part is not perfectly smooth because the material of the last deposited layer is dragged by the nozzle when the internal part of the object is filled. At variance, a better adhesion was observed between the layers of 30CC. As widely reported in the literature [48,49], the interlayer bonding and welding are determined by the diffusion of polymer chains through the interface and their relaxation. The macromolecular interdiffusion leading to successful welding is favored in material showing a predominantly viscous behavior at the welding temperature. In other words, improved interlayer adhesion is expected if the values of the viscous modulus (G″) of the melt are higher than those of the elastic one (G′) in the terminal region [22]. Considering that the extrusion temperature is set at 260 °C and a certain cooling of the material before deposition on the layer below can be estimated, G′ and G″ curves recorded at 200 °C are shown in Figure 5. It can be seen that, for 30CC, the difference between the viscous and elastic component is more pronounced compared to 30T and 3Cl. The same conclusions can be drawn looking at the curves of tanδ as a function of frequency (allowing a representation of the balance between elastic and viscous behavior of samples), as reported in Figure S2. Furthermore, the presence of well-dispersed lamellar fillers in 30T and 3Cl could hamper the interdiffusion of the macromolecular chains between subsequently deposited layers. Otherwise, this phenomenon may not occur in the 30CC system, in which the low-aspect-ratio particles of calcium carbonate interfere to a lesser extent with the macromolecular motion.

### 3.3. Mechanical Properties

**Figure 6:**
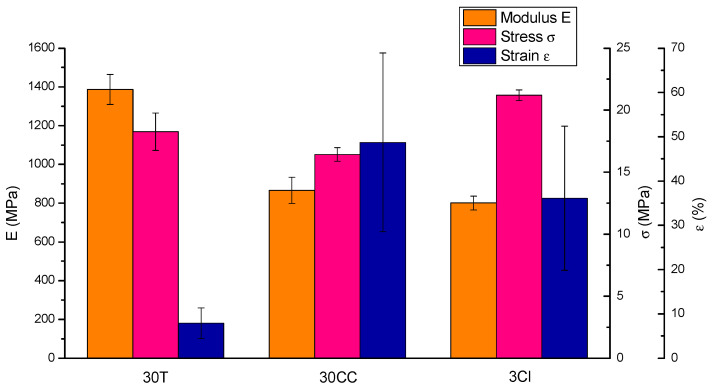
Average values of modulus, stress, and strain of tensile-tested 3D-printed specimens.

The average values and standard deviations of the elastic modulus, maximum tensile stress, and elongation at break are reported in Figure 6. The tensile behavior is clearly influenced by the type and aspect ratio of the embedded filler. The mechanical tests carried out on 30T revealed the highest elastic modulus value (1387 ± 77 MPa) compared to 30CC (865 ± 68 MPa) and 3Cl (801 ± 35 MPa), which instead have quite comparable moduli. As far as elongation at break was concerned, the 30CC formulation showed the highest value (49% ± 20), followed by 3Cl (36% ± 16), and lastly, 30T (8% ± 4). The study by Winter K. et al. found an elastic modulus of about 1300 MPa and an elongation at break of about 10% for a rectangular specimen of PP and 10 wt% of talc tensile tested at 45° to the print direction [26]. The 3Cl specimen finally presents the maximum tensile stress value (21.2 ± 0.4 MPa), followed by 30T (18.3 ± 1.5 MPa) and 30CC (16.4 ± 0.6 MPa). Comparable values were obtained in a study by Milosevic M. et al. in which samples of pre-consumer recycled PP 3D printed with concentric shapes in-fill geometry exhibited a maximum tensile stress equal to about 17 MPa, which becomes 16 and 23 MPa when loaded with 30 wt% hemp fiber and 30 wt% harakeke fiber, respectively [50].

## 4. Conclusions

PP-based composites containing different amounts of micro-sized (i.e., talc, calcium carbonate, and silica) and nano-sized (i.e., nanoclay) fillers were formulated via melt compounding, with the aim of designing materials with optimized characteristics suitable for FFF processing. A preliminary screening of the thermal and rheological properties of the formulated materials allowed the selection of three different formulations, namely composites containing 30 wt% of talc, 30 wt% of calcium carbonate, and 3 wt% of nanoclay, for the production of filaments and the subsequent 3D printing process. The tensile characterization of the 3D-printed specimens demonstrated the possibility of achieving tailored mechanical properties depending on the type of embedded filler. More specifically, the obtained results showed that composites containing talc possess higher stiffness values compared to the other samples, while the introduction of the nanoclay results indicated the obtainment of a more ductile behavior. In all, this study clearly demonstrated that modulable mechanical characteristics can be achieved as a function of the type of embedded filler, allowing for the production of 3D-printed materials with specific characteristics suitable for different fields of application.

## Figures and Tables

**Table 1:** Codes and compositions of prepared materials.

Code	PP [wt%]	Additives [wt%]	Compatibilizer [wt%]
10T	90	10 Talc	-
20T	80	20 Talc	-
30T	70	30 Talc	-
10CC	90	10 Calcium carbonate	-
20CC	80	20 Calcium carbonate	-
30CC	70	30 Calcium carbonate	-
10S	90	10 Silica	-
20S	80	20 Silica	-
30S	70	30 Silica	-
1Cl	98	1 Cloisite 20	1 PP-g-MA
3Cl	94	3 Cloisite 20	3 PP-g-MA

**Table 2:** Filament fabrication parameters for 30T, 30CC, and 3Cl.

	30T	30CC	3Cl
Temperature profile [°C]	210-205-205-200	200-195-195-190	200-195-195-190
Screw speed [rpm]	4.5	3.5	4
Cooling fans [%]	40	70	30

## Data Availability

Data are available upon request.

## Supplementary Materials

The following supporting information can be downloaded at: https://www.mdpi.com/article/10.3390/polym15102263/s1, Figure S1: Al and Si EDS mapping on the SEM image of 3Cl and weight fraction of the detected elements, Figure S2: Tanδ as a function of frequency for PP-based materials. The curves were recorded at 200 °C.
