# Supplementary material for: Optimizing the Rheological and Thermal Behavior of Polypropylene-Based Composites for Material Extrusion Additive Manufacturing Processes
Source: Polymers (Basel). 2023 May 11;15(10):2263. doi: 10.3390/polym15102263 (PMC10224435; doi:10.3390/polym15102263)
Supplement: Supplementary file 1 [file polymers-15-02263-s001.zip › polymers-2382397-supplementary.pdf]

Supplementary Material

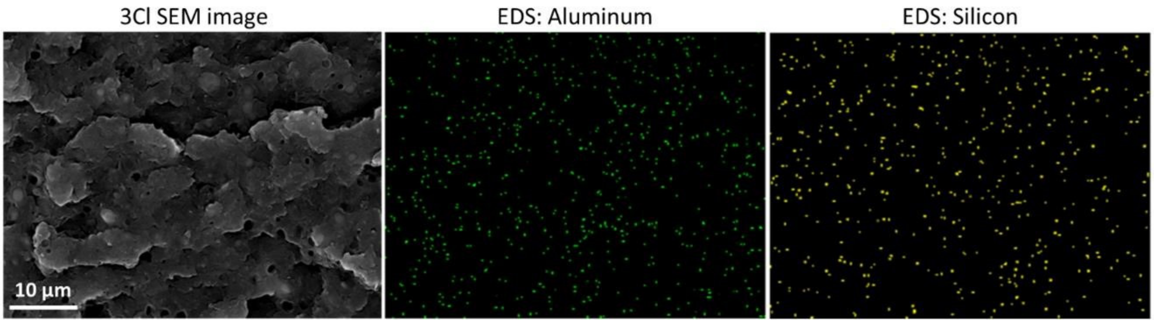

EDS: weight fraction

Element	Wt.%
C	98
Al	1.4
Si	0.6

Figure S1. Al and Si EDS mapping on the SEM image of 3Cl and weight fraction of the detected elements.

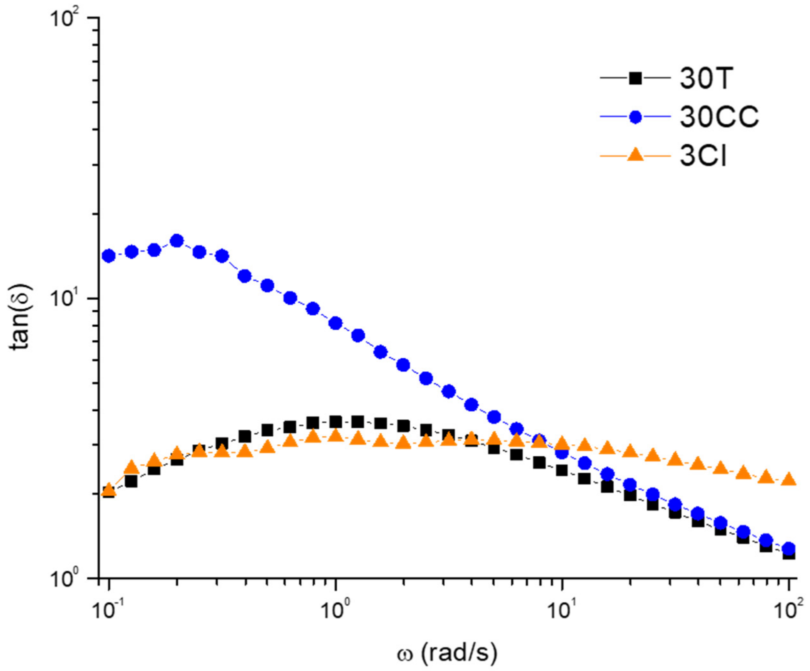

Figure S2. Tanδ as a function of frequency for PP-based materials. The curves were recorded at 200 °C.
